# RGIFE: a ranked guided iterative feature elimination heuristic for the identification of biomarkers

**DOI:** 10.1186/s12859-017-1729-2

**Published:** 2017-06-30

**Authors:** Nicola Lazzarini, Jaume Bacardit

**Affiliations:** ICOS research group, School of Computing Science, Newcastle-upon-Tyne, UK

**Keywords:** Biomarkers, Feature reduction, Knowledge extraction, Machine learning

## Abstract

**Background:**

Current -*omics* technologies are able to sense the state of a biological sample in a very wide variety of ways. Given the high dimensionality that typically characterises these data, relevant knowledge is often hidden and hard to identify. Machine learning methods, and particularly feature selection algorithms, have proven very effective over the years at identifying small but relevant subsets of variables from a variety of application domains, including -omics data. Many methods exist with varying trade-off between the size of the identified variable subsets and the predictive power of such subsets. In this paper we focus on an heuristic for the identification of biomarkers called RGIFE: Rank Guided Iterative Feature Elimination. RGIFE is guided in its biomarker identification process by the information extracted from machine learning models and incorporates several mechanisms to ensure that it creates minimal and highly predictive features sets.

**Results:**

We compare RGIFE against five well-known feature selection algorithms using both synthetic and real (cancer-related transcriptomics) datasets. First, we assess the ability of the methods to identify relevant and highly predictive features. Then, using a prostate cancer dataset as a case study, we look at the biological relevance of the identified biomarkers.

**Conclusions:**

We propose RGIFE, a heuristic for the inference of reduced panels of biomarkers that obtains similar predictive performance to widely adopted feature selection methods while selecting significantly fewer feature. Furthermore, focusing on the case study, we show the higher biological relevance of the biomarkers selected by our approach. The RGIFE source code is available at: http://ico2s.org/software/rgife.html.

**Electronic supplementary material:**

The online version of this article (doi:10.1186/s12859-017-1729-2) contains supplementary material, which is available to authorized users.

## Background

Recent advances in high-throughput technologies yielded an explosion of the amount of -omics data available to scientists for many different research topics. The suffix -omics refers to the collective technologies used to explore the roles, relationships, and actions of the various types of molecules that make up the cellular activity of an organism. Thanks to the continuous cost reduction of bio-technologies, many laboratories nowadays produce large-scale data from biological samples as a routine task. This type of experiments allows the analysis of the relationships and the properties of many biological entities (e.g. gene, proteins, etc.) at once. Given this large amount of information, it is impossible to extract insight without the application of appropriate computational techniques.

One of the major research field in bioinformatics involves the discovery of driving factors, biomarkers, from disease-related datasets where the samples belong to different categories representing different biological or clinical conditions (e.g. healthy vs. disease affected patients). A biomarker is defined as: *“a characteristic that is objectively measured and evaluated as an indicator of normal biological processes, pathogenic processes, or pharmacologic responses to a therapeutic intervention”* [1]. Feature selection is a process, employed in machine learning and statistics, of selecting relevant variables to be used in the model construction. Therefore, the discovery of biomarkers from -omics data can be modelled as a typical feature selection problem. The goal is to identify a subset of features (biomarkers), commonly called *signature*, that can build a model able to discriminate the category (label) of the samples and eventually provide new biological or clinical insights.

Machine learning has been extensively used to solve the problem of biomarkers discovery [2]. Abeel et al. presented a framework for biomarkers discovery in a cancer context based on ensemble methods [3], Wang et al. showed that a combination of different classification and feature selection approaches identifies relevant genes with high confidence [4]. To achieve efficient gene selection from thousands of candidate genes, particle swarm optimisation was combined with a decision tree classifier [5].

Over the years different feature selection methods have been designed, some have been created explicitly to tackle biological problems, others have been conceived to be more generic and can be applied to a broad variety of problems. A common approach for feature selection methods is to rank the attributes based on an importance criteria and then select the top *K* [6]. One of the main drawbacks is that the number *K* of features to be selected needs to be set up-front and determine its exact value is a non-trivial problem. Other methods such as CFS [7] or mRMR (minimum Redundancy Maximum Relevance) [8] are designed to evaluate the goodness of a given subset of variables in relation to the class/output variable. When coupled with a search mechanism (e.g. BestFirst), they can automatically identify the optimal number of features to be selected. A large class of feature selection methods are based on an iterative reduction process. The core of these methods is to iteratively remove the useless feature(s) from the original dataset until a stopping condition is reached. The most well known and used algorithm based on this paradigm is SVM-RFE [9]. This method iteratively repeats 3 steps: 1) trains an SVM classifier, 2) ranks the attributes based on the weights of the classifier and 3) removes the bottom ranked attribute(s). SVM-RFE was originally designed and applied to transcriptomics data, but nowadays it is commonly used in many different contexts. Several approaches have been presented after SVM-RFE [10–12].

In this paper, we present an improved heuristic for the identification of reduced biomarker signatures based on an iterative reduction process called *RGIFE: Ranked Guided Iterative Feature Elimination*. In each iteration, the features are ranked based on their importance (contribution) in the inferred machine learning model. Within its process, RGIFE dynamically removes blocks of attributes rather than in a static (fixed) approach as many of the proposed methods. RGIFE also introduces the concept of *soft-fail*, that is, under certain circumstances, we consider an iteration successful if it suffered a drop in performance within a tolerance level.

This work is an extension of [13] where the heuristic was originally presented. We have thoroughly revisited every aspect of the original work, and in this paper we extend it by: 1) using a different machine learning algorithm to rank the features and evaluate the feature subsets, 2) introducing strategies to reduce the probability of finding a local optimum solution, 3) limiting the stochastic nature of the heuristic, 4) comparing our methods with some well known approaches currently used in bioinformatics 5) evaluating the performance using synthetic datasets and 6) validating the biological relevance of our signatures using a prostate cancer dataset as a case study.

First, we compared the presented version of RGIFE with the original method proposed in [13]. Then, we contrasted RGIFE with five well-known feature extraction methods from both a computational (using synthetic and real-world datasets) and a biological point of view. Finally, using a prostate cancer dataset as a case study, we focused on the knowledge associated with the signature identified by RGIFE. We found that the new proposed heuristic outperforms the original version both in terms of prediction accuracy and number of selected attribute, while being less computationally expensive. When compared with other feature reduction approaches, RGIFE showed similar prediction performance while constantly selecting fewer features. Finally, the analysis performed in the case study showed higher biological (and clinical) relevance of the genes identified by RGIFE when compared with the proposed benchmarking methods. Overall, this work presents a powerful machine-learning based heuristic that when applied to large-scale biomedical data is capable of identifying small sets of highly predictive and relevant biomarkers.

## Methods

### The RGIFE heuristic

A detailed pseudo-code that describes the RGIFE heuristic is depicted in Algorithm 1. RGIFE can work with any (-omics) dataset as long as the samples are associated with discrete classes (e.g. cancer vs. normal) as the signature is identified via the solving of a classification problem. The first step is to estimate the performance of the original set of attributes, this will initially guide the reduction process (line 29). The function RUN_ITERATION() splits the dataset into training and test data by implementing a *k*-fold cross-validation (by default *k*=10) process to assess the performance of the current set of attributes. We opted for a *k*-fold cross-validation scheme, rather than the leave-one-out used in the previous RGIFE version, because of its better performance when it comes to model selection [14]. In here, to describe the RGIFE heuristic, the generic term *performance* is used to refer to how well the model can predict the class of the test samples. In reality, within RGIFE many different measures can be employed to estimate the model performance (accuracy, F-measure, AUC, etc.). The *N* parameter indicates how many times the cross-validation process is repeated with different training/test partitions, this is done in order to minimise the potential bias introduced by the randomness of the data partition. The generated model (classifier) is then exploited to rank the attributes based on their importance within the classification task. Afterwards, the block of attributes at the bottom of the rank is removed and a new model is trained over the remaining data (lines 33–35). The number of attributes to be removed in each iteration is defined by two variables: *blockRatio* and *blockSize*. The former represents the percentage of attributes to remove (that decreases under certain conditions), the latter indicates the absolute number of attributes to remove and is based on the current size of the dataset. Then, if the new performance is equal or better than the reference (line 49), the removed attributes are permanently eliminated. Otherwise, the attributes just removed are placed back in the dataset. In this case, the value of *startingIndex*, a variable used to keep track of the attributes been tested for removal, is increased. As a consequence, RGIFE evaluates the removal of the next *blockSize* attributes, ranked (in the reference iteration) just after those placed back. The *startingIndex* is iteratively increased, in increments of *blockSize*, if the lack of the successive blocks of attributes keeps decreasing the predictive performance of the model. With this iterative process, RGIFE evaluates the importance of different ranked subsets of attributes. Whenever either all the attributes of the current dataset have been tested (i.e. have been eliminated and the performance did not increase), or there has been more than 5 consecutive unsuccessful iterations (i.e. performance was degraded), *blockRatio* is reduced by a fourth (line 44). The overall RGIFE process is repeated while *blockSize* (number of attributes to remove) is ≥ 1.

An important characteristic of RGIFE is the concept of *soft-fail*. After five unsuccessful iterations, if some past trial failed and suffered a “small” drop in performance (one misclassified sample more than the reference iteration) we still consider it as successful (line 40). The reason behind this approach is that by accepting a temporary small degrade in performance, RGIFE might be able to escape from a local optimum and quickly after recover from this little loss in performance. Given the importance of the soft-fail, as illustrated later by the “Analysis of the RGIFE iterative reduction process” section, in this new RGIFE implementation, the searching for the soft-fail is not only performed when five consecutive unsuccessful trials occurs, as in the original version, but it occurs before every reduction of the block size. Furthermore, we extended the iterations that are tested for the presence of a soft-fail. While before only the last five iterations were analysed, now the searching window is expanded up to the most recent between the reference iteration and the iteration in which the last soft-fail was found. Again, this choice was motivated by the higher chance that RGIFE has to identify soft-fails when many unsuccessful iterations occur.

#### Relative block size

One of the main changes introduced in this new version of the heuristic is the adoption of a *relative block size*. The term block size defines the number of attributes that are removed in each iteration. In [13], the 25% of the attributes was initially removed, then, whenever having: all the attributes tested, or five consecutive unsuccessful iterations, the block size was reduced by a fourth. However, our analysis suggested that this policy was prone to get stalled early in the iterative process and prematurely reduce the block size to a very small number. This scenario either slows down the iterative reduction process because successful trials will only remove few attributes (small block size), or it prematurely stops the whole feature reduction process if the size of the dataset in hand becomes too small (few attributes) due to large chunks of attributes being removed (line 33 in Algorithm 1). To address this problem, this new implementation of the heuristic introduces the concept of the relative block size. By using a new variable, *blockRatio*, the number of attributes to be removed is now proportional to the size of the current attribute set being processed, rather than to the original attribute set. While before the values of *blockSize* were predefined (based on the original attribute set), now they vary according to the size of the data being analysed.



#### Parameters of the classifier

RGIFE can be used with any classifier that is able to provide an attribute ranking after the training process. In the current version of RGIFE, we changed the base classifier from BioHEL [15], a rule-based machine learning method based on a genetic algorithm, to a random forest [16], a well-known ensemble-based machine learning method. This is mainly due to reduce the computational cost (see the computational analysis provided in Section 2 of the Additional file 1). We opted for a random forest classifier as it is known for its robustness to noise and its efficiency, so it is ideally suited to tackle large-scale -omics data. The current version of the heuristic is implemented in Python and uses the random forest classifier available in the *scikit-learn* library [17]. In this package, the attributes are ranked based on the *gini impurity*. The feature importance is calculated as the sum over the number of splits (across every tree) that includes the feature, proportionally to the number of samples it splits. Default values for all the parameters of the classifier are used within the heuristic, except for the number of trees (set to 3000 because it provided the best results in preliminary tests not reported here).

#### RGIFE policies

The random forest is a stochastic ensemble classifier, given that each decision tree is built by using a random subset of features. As a consequence, RGIFE inherits this stochastic nature, that is each run of the algorithm results in a potential different optimal subset of features. In addition, the presence of multiple optimal solutions is a common scenario when dealing with high dimensional -omics data [18]. This scenario is addressed by running RGIFE multiple times and using different policies to select the final model (signature):

- *RGIFE-Min*: the final model is the one with the smallest number of attributes
- *RGIFE-Max*: the final model is the one with the largest number of attributes
- *RGIFE-Union*: the final model is the union of the models generated across different executions

In the presented analysis, the signatures were identified from 3 different runs of RGIFE.

### Benchmarking algorithms

We compared RGIFE with five well-known feature selection algorithms: CFS [7], SVM-RFE [9], ReliefF [19], Chi-Square [20] and L1-based feature selection [21]. These algorithms were chosen in order to cover different approaches that can be used to tackle the feature selection problem, each of them employs a different strategy to identify the best subset of features.

**CFS** is a correlation-based feature selection method. By exploiting a best-first search, it assigns high scores to subsets of features highly correlated to the class attribute but with low correlation between each other. Similarly to RGIFE, CFS automatically identifies the best size of the signature.

**SVM-RFE** is a well known iterative feature selection method that employs a backward elimination procedure. The method ranks the features by training an SVM classifier (linear kernel) and discarding the least important (last ranked). SVM-RFE have been successfully applied in classification problems involving -omics datasets.

**ReliefF** is a supervised learning algorithm that considers global and local feature weighting by computing the nearest neighbours of a sample. This method is well employed due to its fast nature as well with its simplicity.

**Chi-Square** is a feature selection approach that computes chi-squared (*χ* ^2^) stats between each non-negative feature and class. The score can be used to select the *K* attributes with the highest values for the chi-squared statistic from the data relative to the classes.

**L1-based feature selection** is an approach based on the L1 norm. Using the L1 norm, sparse solutions (models) are often generated where many of the estimated coefficients (corresponding to attributes) are set to zero. A linear model (a support vector classifier, SVC) penalised with the L1 norm was used to identify relevant attributes [21]. The features with non-zero coefficients in the model generated from the training data were kept and used to filter both the training and the test set. Those were selected because of their importance when predicting the outcome (class label) of the samples.

The L1-based feature selection was evaluated using the *scikit-learn* implementation of the SVC [17], the other benchmarking algorithms were tested with their implementation available in WEKA [22]. Default parameters were used for all the methods, the list of default values are listed in Section 3 of the Additional file 1.

### Datasets

#### Synthetic datasets

To test the ability of RGIFE to identify relevant features, we used a large set of synthetic datasets. The main characteristics of the data are available in Table 1. Different possible scenarios (correlation, noise, redundancy, non-linearity, etc.) were covered using the datasets employed in [23] as a reference (the LED data were not used as they consist of a 10-class dataset that does not reflect a typical biological problem).

**Table 1:** Description of the synthetic datasets used in the experiments

Name	Attributes	Samples	Characteristics
CorrAL [24]	99	32	Corr.; F ≫ S
XOR-100 [24]	99	50	N.L; F ≫ S
Parity3+3 [23]	12	64	NL
Monk3 [25]	6	122	No.
SD1 [26]	4020	75	F ≫ S
SD2 [26]	4040	75	F ≫ S
SD3 [26]	4060	75	F ≫ S
Madelon [27]	500	2400	N.L; No.

*Corr*. stands for correlation, *N.L* indicates nonlinearity, *F ≫ S* is used for datasets where the number of features is higher than the number of samples and *No.* represents noisy data

##### CorrAL

is a dataset with 6 binary features (i.e. *f*_1_,*f*_2_,*f*_3_,*f*_4_,*f*_5_,*f*_6_) where the class value is determined as (*f*_1_∧*f*_2_)∨(*f*_3_∧*f*_4_). The feature *f*_5_ is irrelevant while *f*_6_ is correlated to the class label by 75%. In addition, the data contains 93 irrelevant features randomly added [24].

##### XOR-100

includes 2 relevant and 97 irrelevant (randomly generated) features. The class label consists of the XOR operation between two features: (*f*_1_⊕*f*_2_) [24].

##### Parity3+3

describes the problem where the output is *f*(*x*_1_,…*x*_*n*_)=1 if the number of *x*_*i*_=1 is odd. The *Parity3+3* extends this concept to the parity of three bits and uses a total of 12 attributes [23].

##### Monk3

is a typical problem of the artificial robot domain. The class label is defined as (*f*_5_=3∧*f*_4_=)∨(*f*_5_≠4∧*f*_2_≠3) [25].

##### SD1, SD2 and SD3

are 3-class synthetic datasets where the number of features (around 4000) is higher than the number of samples (75 equally split into 3 classes) [26]. They contain both full class relevant (FCR) and partial class relevant (PCR) features. FCR attributes serve as biomarkers to distinguish all the cancer types (labels), while PCRs discriminate subsets of cancer types. SD1 includes 20 FCRs and 4000 irrelevant features. The FCR attributes are divided into two groups (attributes) of ten, genes in the same group are redundant. The optimal solution consists of any two relevant features coming from different groups. SD2 includes 10 FCRs, 30 PCRs and 4000 irrelevant attributes. The relevant genes are split into groups of ten; the optimal subset should combine one gene from the set of FCRs and three genes from the PCRs, each one from a different group. Finally, SD3 contains only 60 PCRs and 4000 irrelevant features. The 60 PCRs are grouped by ten, the optimal solution consists of six genes, one from each group. Collectively we will refer to SD1, SD2 and SD3 as the SD datasets.

##### Madelon

is a dataset used in the NIPS’2003 feature selection challenge [27]. The relevant features represent the vertices of a 5-dimensional hypercube. 495 irrelevant features are added either from a random gaussian distribution or multiplying the relevant features by a random matrix. In addition, the samples are distorted by flipping labels, shifting, rescaling and adding noise. The characteristic of Madelon is the presence of many more samples (2400) than attributes (500).

All the presented datasets were provided by the authors of [23]. In addition, we generated two-biological conditions (control and case) synthetic microarray datasets using the *madsim* R package [28]. Madsim is a flexible microarray data simulation model that creates synthetic data similar to those observed with common platforms. Twelve datasets were generated using default parameters but varying in terms of number of attributes (5000, 10,000, 20,000 and 40,000) and percentage of up/down regulated genes (1%, 2% and 5%). Each dataset contained 100 samples equally distributed in controls and cases. Overall, *madsim* was ran with the following parameters: *n*={5000,10,000,20,000,40,000} *p* *d* *e*={0.01,0.02,0.05} and *m*_1_=*m*_2_=50.

#### Real-world datasets

We used ten different cancer-related transcriptomics datasets to validate our approach (see Table 2). These datasets represent a broad range of characteristics in terms of biological information (different types of cancers), number of samples and number of attributes (genes).

**Table 2:** Description of the real-world datasets used in the experiments

Name	Attributes	Samples
Prostate-Sboner ([58])	6144	281
Dlbcl ([59])	7129	77
CNS ([60])	7129	60
Leukemia ([61])	7129	72
Prostate-Singh ([30])	12600	102
AML ([40])	12625	54
Colon-Breast ([62])	22283	52
Bladder ([63])	43148	166
Breast ([39])	47293	128
Pancreas ([64])	54675	78

### Experimental design

While CFS and the L1-based feature selection automatically identify the optimal subset of attributes, the other algorithms require to specify the number of attributes to retain. To obtain a fair comparison, we set this value to be equal to the number of features selected by the RGIFE’s Union policy (as by definition it generates the largest signature among the policies). For all the tested methods, default parameter values were used for the analysis of both synthetic and real-world datasets.

#### Relevant features identification

We used the scoring measure proposed by Bolon et. al [23] to compute the efficacy of the different feature selection methods in identifying important features from synthetic data. The *Success Index* aims to reward the identification of relevant features and penalise the selection of irrelevant ones:

$$Success\,Index = 100\times\left(\frac{R_{s}}{R_{t}}-\alpha\frac{I_{s}}{I_{t}}\right) \; ; \alpha=min\left\{ \frac{1}{2},\frac{R_{t}}{I_{t}}\right\} $$

where *R*_*s*_ and *R*_*t*_ are the number of relevant features selected and the total number of relevant features. Similarly, *I*_*s*_ and *I*_*t*_ represent the number of selected and the total number of irrelevant features.

#### Predictive performance validation

The most common metric to assess the performance of a feature selection method is by calculating the accuracy when predicting the class of the samples. The accuracy is defined as the rate of correctly classified samples over the total number of samples. A typical *k*-fold cross-validation scheme randomly divides the dataset *D* in *k* equally-sized disjoint subsets *D*_1_,*D*_2_,…,*D*_*k*_. In turn, each fold is used as test set while the remaining *k*−1 are used as training set. A *stratified* cross-validation aims to partition the dataset into folds where the original distribution of the classes is preserved. However, the stratified cross-validation does not take into account the presence of clusters (similar samples) within each class. As observed in [14], this might result in a distorted measure of the performances. Dealing with transcriptomics datasets that have a small number of observations (e.g. CNS only has 60 samples), the distortion in performances can be amplified. In order to avoid this problem, we adopted the DB-SCV (Distributed-balanced stratified cross-validation) scheme proposed in [29]. DB-SCV is designed to assign close-by samples to different folds, so each fold will end up having enough representatives of every possible cluster. We modified the original DB-SCV scheme so that the residual samples are randomly assigned to the folds. A dataset with *m* samples, when using a *k*-fold cross-validation scheme has in total (*m* mod *n*) residual samples. By randomly assigning the residual samples to the folds, rather than sequentially as in the proposed DB-SCV, we obtain a validation scheme that can better estimate the predictive performance of unseen observations.

We used a 10-fold DB-SCV scheme for all the feature selection methods by applying them to the training sets and mirroring the results (filtering the selected attributed) to the test sets. The 10-fold DB-SCV scheme was also employed in RGIFE (line 17–18) with *N*=10. The model performance within RGIFE was estimated using the accuracy metric (by averaging the accuracy values across the folds of the 10-fold DB-SCV).

#### Validation of the predictive performance of identified signatures

The performances of the biomarker signatures identified by different methods were assessed using four classifiers: random forest (RF), gaussian naive bayes (GNB), SVM (with a linear kernel) and K-nearest neighbours (KNN). Each classifier uses different approaches and criteria to predict the label of the samples, therefore we test the predictive performance of each method in different classification scenarios. We used the *scikit-learn* implementations for all the classifiers with default parameters, except for the depth of the random forest trees, which was set to 5 in order to avoid overfitting (considering the small number of attributes in each signature). The stochastic nature of RF was addressed by generating ten different models for each training set and defining the predicted class via a majority vote.

#### Biological significance analysis of the signatures

We validated the biological significance of the signatures generated by different methods using the *Prostate-Singh* [30] dataset as a case study. The biological relevance was assessed studying the role of the signatures’ genes: in a cancer-related context, in a set of independent prostate-related datasets and finally in a protein-protein interaction network (PPI).

##### Gene-disease associations

In order to assess the relevance of the signatures within a cancer-related context, we checked whether their genes were already known to be associated with a specific disease. From the literature, we retrieved the list of genes known to be associated with prostate cancer. We used two sources for the information retrieval: *Malacards* (a meta-database of human maladies consolidated from 64 independent sources) [31] and the union of four manually curated databases (OMIM [32], Orphanet [33], Uniprot [34] and CTD [35]). We checked the number of disease-associated genes included in the signatures and we calculated *precision*, *recall* and *F-measure*. The *precision* is the fraction of genes that are associated with the disease, while the *recall* is the fraction of disease-associated genes (from the original set of attributes) included in the signature. Finally, the *F-measure* is calculated as the harmonic mean of *precision* and *recall*.

##### Gene relevance in independent datasets

We searched the public prostate cancer databases to verify if the genes, selected by the different methods, are relevant also in data not used for the inference of the signatures. We focused on eight prostate cancer related datasets available from the cBioPortal for Cancer Genomics [36]: *SUC2, MICH, TCGA, TGCA 2015, Broad/Cornell 2013, MSKCC 2010, Broad/Cornell 2012* and *MSKCC 2014*. We checked if the selected genes were genomically altered in the samples of the independent data. For each method and for each independent dataset, we calculated the average fraction of samples with genomic alterations for the selected biomarkers. In order to consider the different size of each signature, the values have been normalised across methods (i.e. divided by the number of selected genes).

##### Signature induced network

A part of the biological confirmation of our signatures involved its analysis in a network context. It was interesting to check if the genes selected by RGIFE interact with each other. To address this question, a signature induced network was generated from a PPI network by aggregating all the shortest paths between all the genes in the signature. If multiple paths existed between two genes, the path that overall (across all the pairs of genes) was the most used was included. The paths were extracted from the PPI network employed in [37] that was assembled from 20 public protein interaction repositories (BioGrid, IntAct, I2D, TopFind, MolCon, Reactome-FIs, UniProt, Reactome, MINT, InnateDB, iRefIndex, MatrixDB, DIP, APID, HPRD, SPIKE, I2D-IMEx, BIND, HIPPIE, CCSB), removing non-human interactions, self-interactions and interactions without direct experimental evidence for a physical association.

## Results

### Comparison of the RGIFE predictive performance with the original heuristic

**Fig. 1:**
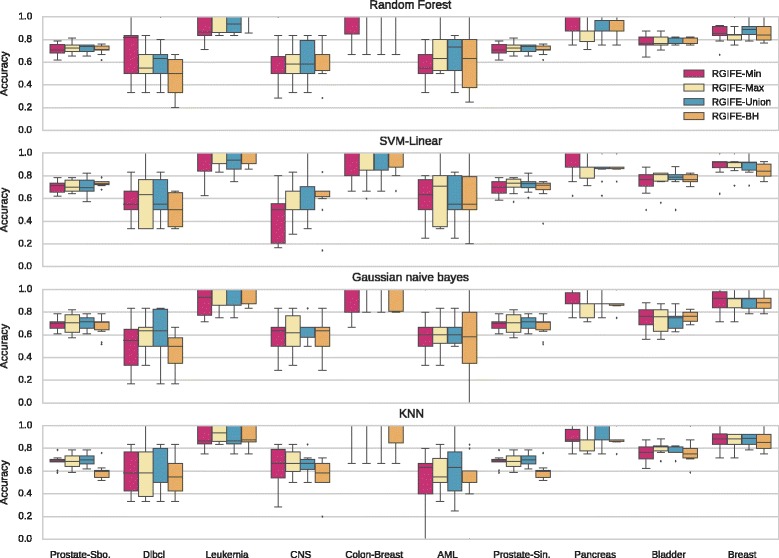
Distribution of the accuracies, calculated using a 10-fold cross-validation, for different RGIFE policies. Each subplot represents the performance, obtained with ten different datasets, assessed with four classifiers

The first natural step for the validation of the new RGIFE was to compare it to its original version, in here named RGIFE-BH after the core machine learning algorithm used within (BioHEL). We compared the predictive performance of the two methods by applying them to the training sets (originated from a 10-fold cross-validation) in order to identify small signatures that are then used to filter the test sets before the prediction of the class labels. In Fig. 1 we show the distribution of accuracies obtained using the ten datasets presented in Table 2. The predictive performance was assessed with four different classifiers. The accuracy of RGIFE-BH is calculated as the average of the accuracies obtained over 3 runs of RGIFE-BH (same number of executions employed to identify the final models with the new RGIFE policies). Across different datasets and classifiers, RGIFE-BH performed basically similar or worse than the new proposed policies based on a random forest. To establish whether the difference in performance was statistically significant, we employed the Friedman rank based test followed by a Nemenyi post-hoc correction. This is a well-known approach in the machine learning community when it comes to the comparison of multiple algorithms over multiple datasets [38]. The ranks, for all the tested classifiers, are provided in Table 3. The attributes selected by RGIFE-BH performed quite well when using a random forest, while for the remaining tested classifiers the performance were generally low. In particular, RGIFE-BH obtained statistically significant worse results (confidence level of 0.05), compared with RGIFE-Union, when analysed with the KNN classifier.

**Table 3:** Average performance ranks obtained by each RGIFE policy across the ten datasets and using four classifiers

Classifier	RGIFE-Min	RGIFE-Max	RGIFE-Union	RGIFE-BH
Random Forest	3.15 (4)	2.60 (3)	**1.85 (1)**	2.40 (2)
SVM-Linear	3.10 (4)	**1.60 (1)**	2.40 (2)	2.90 (3)
Gaussian naive bayes	2.70 (4)	2.65 (3)	**1.75 (1)**	2.90 (4)
KNN	2.70 (3)	2.20 (2)	**1.80 (1)**	3.30 (4)*

The highest ranks are shown in bold

* indicates statistically worse performance

It might be tempting to associate the better performance of the new heuristic with the usage of a better base classifier. However, this is not the case as, when tested with a standard 10-fold cross-validation (using the presented ten transcriptomics datasets with the original set of attributes), random forest and BioHEL obtained statistically equivalent accuracies (Wilcoxon rank-sum statistic). In fact, on average, the accuracy associated with the random forest was only higher by 0.162 when compared to the performance of BioHEL. The accuracies obtained by the methods when classifying the samples using the original set of attributes is available in Section 1 of the Additional file 1.

**Fig. 2:**
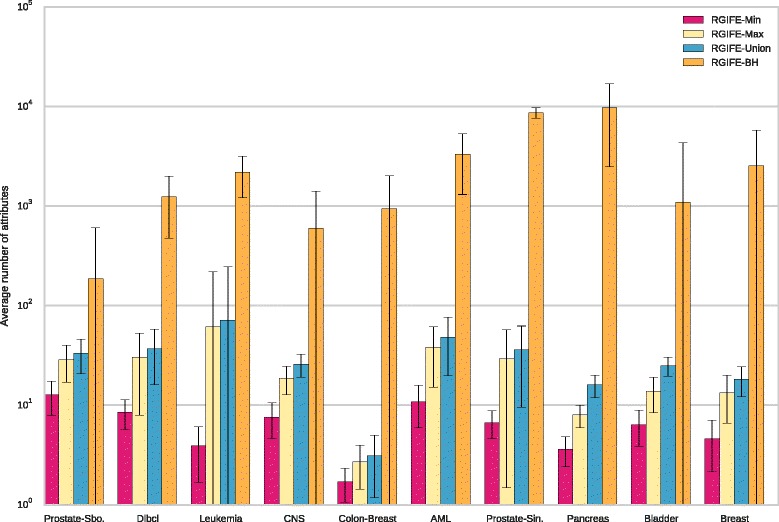
Comparison of the number of selected attributes by different RGIFE policies. For each dataset is reported the average number of attributes obtained from the 10-fold cross-validation together with the standard deviation

In addition, we also compared the number of attributes selected by different RGIFE policies when using different datasets. Figure 2 provides the average number of attributes selected, across the folds of the cross-validation, by the original and the new proposed version of RGIFE. The number of attributes represented for RGIFE-BH is the result of an average across its three different executions. In each of the analysed dataset, the new RGIFE was able to obtain a smaller subset of predictive attributes while providing higher accuracies. The better performance, in terms of selected attributes, of the new heuristic is likely the result of the less aggressive reduction policy introduced by the relative block size. By removing chunks of attributes whose sizes are proportional to the volume of the dataset being analysed, the heuristic is more prone to improve the predictive performance across iterations. Moreover, by guaranteeing more successful iterations, a smaller set of relevant attributes can be identified. The difference is particularly evident when RGIFE was applied to the largest datasets (in Fig. 2 the datasets are sorted by increasing size).

Finally, as expected, the replacement of BioHEL with a faster classifier drastically reduces the overall computational time required by RGIFE. In Section 2 of the Additional file 1 is available a complete computational analysis of every method tested for this paper.

### Identification of relevant attributes in synthetic datasets

A large set of synthetic data was used to assess the ability of each method to identify relevant features in synthetic datasets. The *Success Index* is used to determine the success of discarding irrelevant features while focusing only on the important ones. Table 4 reports a summary of this analysis, the values correspond to the average Success Index obtained when using a 10-fold cross-validation. The higher the Success Index, the better the method, 100 is its maximum value. In Section 4 of Additional file 1 are reported the accuracies of each method using four different classifiers.

**Table 4:** Average Success Index calculated using a 10-fold cross-validation

Dataset	RGIFE-Min	RGIFE-Max	RGIFE-Union	CFS	ReliefF	SVM-RFE	Chi-Square	L1
CorrAL	59.93	77.11	**87.07**	84.57	72.04	64.53	82.06	84.11
XOR-100	**89.99**	79.88	89.88	24.72	49.86	14.84	9.84	79.30
Parity3+3	44.44	44.44	**76.67**	-5.93	**76.67**	-15.93	-8.52	5.56
Monk3	**84.17**	**84.17**	**84.17**	62.50	**84.17**	N/A	59.17	73.33
Madelon	59.98	77.98	87.97	17.99	89.97	23.97	39.97	**99.01**
Average	67.70	72.72	**85.15**	36.77	74.54	21.85	36.50	68.26

The last row reports the average values across the five datasets. The highest indexes are shown in bold. N/A is used for SVM-RFE when analysing the Monk3 dataset as the method cannot deal with categorical attributes

RGIFE-Union is the method with the highest average Success Index, followed by RGIFE-Max and ReliefF. The Union policy clearly outperforms the other methods when analysing the *Parity3+3* and the *XOR-100* datasets. Overall, SVM-RFE seemed unable to discriminate between relevant and irrelevant features. Low success was also observed for CFS and Chi-Square. For the analysis of the SD datasets [26] we report measures that are more specific for the problem. The SD data are characterised by the presence of relevant, redundant and irrelevant features. For each dataset, Table 5 includes the average number of: selected features, features within the optimal subset, irrelevant and redundant features.

**Table 5:** Results of the SD datasets analysis

Dataset	Metrics	RGIFE-Min	RGIFE-Max	RGIFE-Union	CFS	ReliefF	SVM-RFE	Chi-Square	L1
SD1	Selected	113.3	253.6	289.5	24.3	289.5	289.5	289.5	144.2
	OPT(2)	0.2	0.8	0.9	1.5	2.0	2.0	1.7	2.0
	Redundant	0.0	2.7	2.7	0.3	9.0	8.7	5.4	5.3
	Irrelevant	114.1	248.4	284.2	23.1	270.5	271	278.5	132.6
SD2	Selected	103.4	279.7	319.4	23.1	319.4	319.4	319.4	137.1
	OPT(4)	0.6	1.1	1.2	2.7	3.9	4.0	2.8	4.0
	Redundant	0.6	2.4	2.6	0.1	9	8.9	3.6	3.5
	Irrelevant	102.6	271.4	310.4	20.7	281.4	281.4	301.8	117.9
SD3	Selected	114.6	284.3	337.3	24.4	337.3	337.3	337.3	143.4
	OPT(6)	1.0	2.6	3.8	3.4	4.8	4.2	3.5	6.0
	Redundant	1.0	4.1	6.1	0.1	9.0	4.0	7.4	3.5
	Irrelevant	113.2	267.8	312.9	21.1	292.0	306.6	309.2	119.2

The values are averaged from a 10-fold cross-validation. OPT(x) indicates the average number of selected features within the optimal subset

The L1-based feature selection was the only method always able to select the minimum number of optimal features, however it also picked a large number of irrelevant features. On the other hand, CFS was capable of avoiding redundant and irrelevant features while selecting a high number of optimal attributes. ReliefF, SVM-RFE and Chi-Square performed quite well for SD1 and SD2, but not all the optimal features were identified in SD3. The RGIFE policies performed generally poorly on the SD datasets. Among the three policies, RGIFE-Union selected the highest number of optimal features (together with a large amount of irrelevant information). Despite that, the number of redundant features was often lower than methods which selected more optimal attributes. Interesting, when analysing the accuracies obtained by each method (reported in Table S2 of Additional file 1), we noticed that the attributes selected by RGIFE-Union, although not completely covering the optimal subsets, provide the best performance for SD2 and SD3 (with random forest and GNB classifier). Finally, Table 6 shows the results from the analysis of the data generated with *madsim* [28]. The values have been averaged from the results of the data containing 1, 2 and 5% of up/down regulated genes. Differently from the SD datasets, there is not an optimal subset of attributes, therefore we report only the average number of relevant and irrelevant (not up/down-regulated genes) features. The accuracies of each method (available in Table S3 of Additional file 1) were constantly equal to 1 for most of the methods regardless the classifier used to calculate them. Exceptions are represented by RGFE-Max, RGIFE-Min and Chi-Square. All the RGIFE policies performed better than CFS and L1 in terms of relevant selected attributes. Few up/down regulated attributes, compared with the dozens of the other two methods, were enough to obtain a perfect classification. In addition, RGIFE never used irrelevant genes in the proposed solutions. The other methods, whose number of selected attributes was set equal to that used by RGIFE-Union, performed equally well.

**Table 6:** Results of the *madsim* datasets analysis

Attributes	Metric	RGIFE-Min	RGIFE-Max	RGIFE-Union	CFS	ReliefF	SVM-RFE	Chi-Square	L1
5 000	Rel.	1.0	1.2	2.7	54.9	2.7	2.7	2.7	18.4
	Irr.	0.0	0.0	0.0	0.3	0.0	0.0	0.0	0.0
10 000	Rel.	1.0	1.5	3.1	68.4	3.1	3.1	3.1	22.1
	Irr.	0.0	0.0	0.0	0.0	0.0	0.0	0.0	0.0
20 000	Rel.	1.0	1.3	3.2	96.4	3.2	3.2	3.2	29.2
	Irr.	0.0	0.0	0.0	0.0	0.0	0.0	0.0	0.0
40 000	Rel.	1.0	1.5	3.5	133.8	3.5	3.5	3.5	28.2
	Irr.	0.0	0.0	0.0	0.0	0.0	0.0	0.0	0.0

The values are averaged from the analysis of data containing 1%, 2% and 5% of up/down regulated genes(). For each set of data are reported the average number of relevant (up/down regulated) and irrelevant attributes obtained from a 10-fold cross-fold validation

Overall, the analysis completed using synthetic datasets highlighted the ability of RGIFE, in particular of RGIFEUnion, to identify important attributes from data with different characteristics (presence of noise, nonlinearity, correlation, etc.). Good performance was also reached from data similar to microarray datasets (madsim). On the other hand, the SD datasets led to unsatisfactory RGIFE results. This can be attributed to the low number of samples (only 25) available for each class that can generate an unstable internal performance evaluation (based on a 10-fold cross-validation) of the RGIFE heuristic.

### Comparison of the RGIFE predictive performance with other biomarkers discovery methods

Having established the better performance provided by the presented heuristic compared with its original version, and encouraged by the results obtained using synthetic datasets, we evaluated RGIFE analysing -omics data. For each dataset and base classifier, we calculated the accuracy of the biomarker signatures generated by each method and we ranked them in ascending order (the higher the ranking, the higher the accuracy). In Table 7 we report all the accuracies and the ranks (in brackets), the last column shows the average rank across the datasets for each method. With three out of four classifiers, our approach was the first ranked (once RGIFE-Max, twice RGIFE-Union). ReliefF was the first ranked when evaluated with random forest (RF), while it performed quite poorly when using SVM. Similarly, RGIFE-Max was first and second ranked respectively with SVM and KNN, while it was the second and the third-worse for RF and GNB. Overall, the best RGIFE policy appears to be RGIFE-Union being ranked as first when tested with KNN and GNB. Conversely, RGIFE-Min performed quite badly across classifiers.

**Table 7:** Accuracies and ranks (in brackets) obtained by each method across the ten datasets using four classifiers

Class.	Method	Prostate-Sbo.	Dlbcl	Leukemia	CNS	Colon-Breast	AML	Prostate-Sin.	Pancreas	Bladder	Breast	Avg. Rank
RF	RGIFE-Union	0.723 (6)	0.643 (3)	0.927 (7)	0.617 (5)	**0.947 (3)**	0.667 (3)	0.923 (3)	0.898 (3.5)	0.794 (4)	0.884 (2.5)	4.00 (4)
	RGIFE-Max	0.727 (4)	0.573 (7)	0.940 (5.5)	0.600 (6)	**0.947 (3)**	**0.680 (1)**	0.913 (6.5)	0.859 (8)	0.782 (6)	0.844 (8)	5.50 (6)
	RGIFE-Min	0.712 (8)	**0.680 (1)**	0.886 (8)	0.589 (7)	0.927 (7)	0.577 (8)	0.884 (8)	**0.900 (1.5)**	0.770 (8)	0.851 (7)	6.35 (8)
	CFS	**0.741 (1)**	0.627 (4)	0.957 (2.5)	0.622 (4)	**0.947 (3)**	0.597 (7)	0.922 (5)	0.886 (6)	0.800 (3)	0.869 (6)	4.15 (5)
	SVM-RFE	0.733 (2)	0.623 (5)	0.944 (4)	0.668 (3)	0.887 (8)	0.675 (2)	0.923 (3)	0.898 (3.5)	**0.819 (1)**	0.877 (4)	3.55 (2)
	ReliefF	0.726 (5)	0.577 (6)	**0.961 (1)**	0.681 (2)	0.930 (6)	0.633 (5)	**0.932 (1)**	**0.900 (1.5)**	0.800 (2)	**0.892 (1)**	**3.50 (1)**
	Chi-Square	0.716 (7)	0.660 (2)	0.940 (5.5)	0.520 (8)	**0.947 (3)**	0.622 (6)	0.913 (6.5)	0.886 (6)	0.776 (7)	0.870 (5)	5.60 (7)
	L1	0.730 (3)	0.520 (8)	0.957 (2.5)	**0.684 (1)**	**0.947 (3)**	0.650 (4)	0.923 (3)	0.886 (6)	0.788 (5)	0.884 (2.5)	3.80 (3)
SVM	RGIFE-Union	0.709 (4)	0.523 (4.5)	0.917 (6)	0.565 (4)	0.927 (4)	0.633 (4)	0.895 (5)	0.861 (6)	0.757 (5)	0.892 (3)	4.55 (4)
	RGIFE-Max	**0.716 (1)**	0.573 (2)	0.957 (3.5)	0.572 (3)	**0.947 (1.5)**	0.617 (5)	0.915 (2)	0.873 (5)	0.775 (2)	0.876 (5)	**3.00 (1)**
	RGIFE-Min	0.694 (6)	0.567 (3)	0.908 (7)	0.421 (8)	0.907 (5.5)	0.585 (7)	0.852 (8)	0.875 (4)	0.744 (6)	**0.894 (1.5)**	5.60 (8)
	CFS	0.712 (3)	0.523 (4.5)	0.961 (2)	0.546 (5)	0.943 (3)	0.638 (3)	**0.952 (1)**	**0.911 (1)**	0.770 (3.5)	0.832 (8)	3.40 (2)
	SVM-RFE	0.644 (8)	0.500 (6)	0.942 (5)	0.699 (2)	0.850 (7)	0.500 (8)	0.894 (6)	0.848 (8)	0.770 (3.5)	**0.894 (1.5)**	5.50 (7)
	ReliefF	0.690 (7)	0.407 (8)	0.886 (8)	0.535 (6)	0.747 (8)	0.590 (6)	0.904 (4)	0.900 (2)	**0.795 (1)**	0.886 (4)	5.40 (6)
	Chi-Square	0.705 (5)	**0.603 (1)**	0.957 (3.5)	0.462 (7)	**0.947 (1.5)**	**0.652 (1)**	0.893 (7)	0.857 (7)	0.739 (8)	0.869 (6)	4.70 (5)
	L1	0.716 (2)	0.490 (7)	**0.988 (1)**	**0.746 (1)**	0.907 (5.5)	0.650 (2)	0.913 (3)	0.896 (3)	0.740 (7)	0.851 (7)	3.85 (3)
GNB	RGIFE-Union	0.701 (2)	**0.623 (1)**	0.932 (5)	0.650 (3)	**0.963 (2.5)**	0.627 (4)	0.922 (4)	0.887 (5)	0.733 (6)	0.884 (4)	**3.65 (1)**
	RGIFE-Max	0.698 (3)	0.590 (3)	0.932 (5)	0.620 (5)	**0.963 (2.5)**	0.610 (6.5)	0.922 (4)	0.846 (8)	0.727 (7)	0.876 (6)	5.00 (7)
	RGIFE-Min	0.691 (5)	0.503 (5)	0.890 (8)	0.589 (7)	0.907 (6.5)	0.617 (5)	0.895 (7)	0.900 (2)	0.751 (4)	0.900 (3)	5.25 (8)
	CFS	0.690 (6)	0.520 (4)	**0.973 (1)**	0.665 (2)	0.927 (5)	0.650 (2)	**0.932 (1.5)**	0.871 (7)	0.740 (5)	0.870 (7)	4.05 (3)
	SVM-RFE	0.655 (7)	0.450 (8)	0.958 (3)	0.626 (4)	0.873 (8)	0.593 (8)	0.912 (6)	0.898 (3.5)	**0.807 (1)**	**0.907 (1)**	4.95 (6)
	ReliefF	0.616 (8)	0.473 (7)	0.919 (7)	0.570 (8)	0.907 (6.5)	0.633 (3)	**0.932 (1.5)**	0.898 (3.5)	0.764 (2)	0.901 (2)	4.85 (5)
	Chi-Square	0.694 (4)	0.593 (2)	0.932 (5)	0.620 (6)	**0.963 (2.5)**	0.610 (6.5)	0.922 (4)	**0.925 (1)**	0.727 (8)	0.878 (5)	4.40 (4)
	L1	**0.719 (1)**	0.500 (6)	0.971 (2)	**0.746 (1)**	**0.963 (2.5)**	**0.655 (1)**	0.885 (8)	0.886 (6)	0.753 (3)	0.855 (8)	3.85 (2)
KNN	RGIFE-Union	**0.698 (1)**	0.593 (2)	0.901 (7.5)	0.665 (4)	**0.947 (2.5)**	0.602 (3)	0.894 (2.5)	**0.911 (1.5)**	0.788 (3)	0.876 (6)	**3.30 (1)**
	RGIFE-Max	0.684 (3)	**0.597 (1)**	0.927 (3)	0.670 (3)	**0.947 (2.5)**	0.582 (5)	0.893 (4.5)	0.861 (8)	**0.806 (1)**	0.862 (8)	3.90 (2)
	RGIFE-Min	0.684 (4)	0.587 (3)	0.901 (7.5)	0.635 (5)	**0.947 (2.5)**	0.528 (8)	**0.903 (1)**	0.886 (5.5)	0.758 (8)	0.876 (6)	5.05 (7)
	CFS	0.669 (6)	0.407 (8)	0.917 (5.5)	0.587 (8)	0.910 (6)	0.580 (6)	0.884 (6)	**0.911 (1.5)**	0.776 (5)	0.876 (6)	5.80 (8)
	SVM-RFE	0.662 (7)	0.523 (7)	0.946 (2)	**0.771 (1)**	0.847 (8)	0.562 (7)	0.875 (7)	0.898 (3.5)	0.801 (2)	**0.892 (1)**	4.55 (4.5)
	ReliefF	0.698 (2)	0.553 (5)	0.917 (5.5)	0.615 (7)	0.907 (7)	0.630 (2)	0.894 (2.5)	0.898 (3.5)	0.783 (4)	0.884 (2)	4.05 (3)
	Chi-Square	0.680 (5)	0.537 (6)	0.919 (4)	0.618 (6)	**0.947 (2.5)**	0.595 (4)	0.893 (4.5)	0.873 (7)	0.770 (6)	0.877 (3)	4.80 (6)
	L1	0.645 (8)	0.570 (4)	**0.973 (1)**	0.698 (2)	0.927 (5)	**0.713 (1)**	0.825 (8)	0.886 (5.5)	0.769 (7)	0.876 (4)	4.55 (4.5)

The highest accuracies and ranks are shown in bold. The last column reports the average ranks across the datasets for each method, in brackets are shown the absolute ranks. The accuracies are rounded to the third decimal but the ranks are based on higher precision. *RF* random forest, *KNN* K-nearest neighbour and *GNB* Gaussian Naive Bayes

In order to statistically compare the performances of the methods, we used again the Friedman test with the corresponding Nemenyi post-hoc test. In all the four scenarios there was no statistical difference in the performances of the tested methods. The only exception was ReliefF (first ranked) that statistically outperformed RGIFE-Min when using random forest (confidence level of 0.05). According to these results, we can conclude that the presented approach has predictive performances comparable to the evaluated well-established methods.

### Analysis of the signatures size

**Fig. 3:**
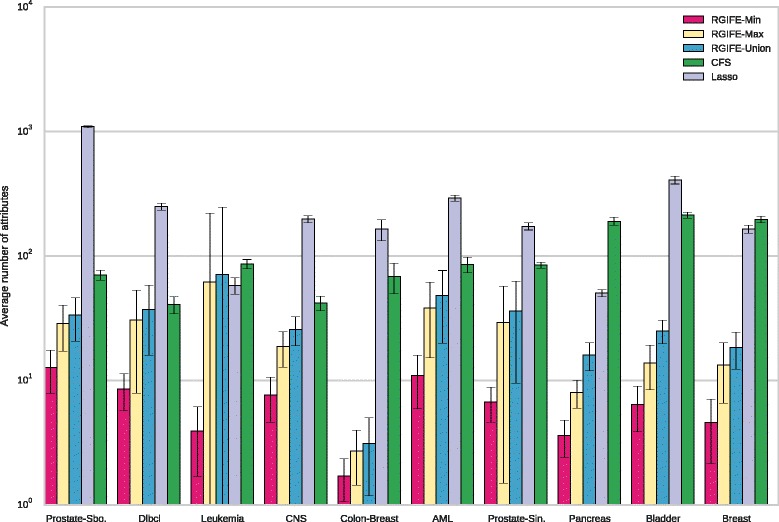
Comparison of the number of selected attributes by the RGIFE policies, CFS and the L1-based feature selection. For each dataset is reported the average number of attributes obtained from the 10-fold cross-validation together with the standard deviation

We compared the size (number of the selected attributes) of the signatures generated by our policies, CFS and L1-based feature selection. With methods such as ReliefF or SVM-RFE, the comparison is meaningless because the number of the selected features is a parameter that needs to be set up-front. The results, dataset by dataset, are shown in Fig. 3. Each bar represents the average number of chosen features across the ten training sets of the cross-validation process described in the “Predictive performance validation” section. There is a clear and remarkable difference in the number of selected attributes by our approach and the other two methods; this is extreme in datasets such as *Colon-Breast* and *Pancreas*. The L1-based feature selection performed quite badly when applied to the smallest dataset (*Prostate-Sboner*). A large standard deviation can be noticed in the number of selected features by RGIFE-Union for the *Leukemia* dataset. This is due to a large signature (around 500 attributes) identified by our approach. The cause of this large number relies on an early stopping condition reached by RGIFE (the block size was reduced too early due to the impossibility to improve the performance of the reference iteration). As expected, the best performing policy is RGIFE-Min. When applying the Friedman test to the average signature size of the methods, RGIFE-Min and RGIFE-Max were statistically better than CFS and the L1-based approach with a confidence level of 0.01. Moreover, RGIFE-Min performed statistically better than RGIFE-Union. Although the Union policy did not statistically outperform the other two methods, the results in Fig. 3 show how it consistently selected fewer features.

### Analysis of the RGIFE iterative reduction process

**Fig. 4:**
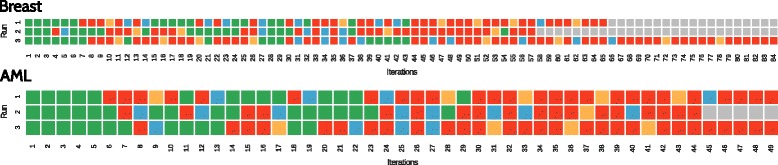
Result of each iteration during the iterative feature elimination process when applied to two datasets (Breast and AML) for 3 different runs of RGIFE. *Green* and *blue* equal or better performance than the reference iteration. *Green* is used when the removed attributes were the bottom ranked, otherwise blue is employed. *Red* represents worse performance, *yellow* shows the identification of a soft-fail. The last *non-grey square* indicates the last iteration of the RGIFE run

RGIFE is an iterative reduction heuristic where each iteration ends with two possible outputs: the performance is either better/equal, compared to the reference iteration, or worse. Given this scenario, it is possible to graphically visualise the behaviour of the whole reduction process. In Fig. 4 it is illustrated the application of RGIFE to two datasets: *Breast* [39] and *AML* [40]. The plot shows the reduction process generated from 3 different runs of RGIFE when applied to the whole dataset to obtain the signature. A different colour represents a different output for the iteration: green and blue show an improvement (or equality) of the performance, blue is used when the removed attributes had not the lowest attribute importance (were not the bottom ranked). Red indicates a decrease in predictive performance, while a yellow square marks the identification of a previous soft-fail (a past iteration that suffered a small drop in performance). RGIFE looks for soft-fails whenever all the attributes have been tested (e.g. iteration 9 on Run 1 for *AML*) or there are 5 consecutive decreases in performance (e.g. iteration 36 on Run 3 for *AML*). Figure 4 shows the presence of several soft-fail iterations, this is likely the results of the new strategies introduced to let the heuristic working with smaller data. In fact, differently than the original version, RGIFE now additionally performs a search for soft-fails before the block size is reduced. Furthermore, the iterations searched for the presence of a soft-fails are not anymore limited to the past five trials but are extended up to the reference one (or the last trial in which a soft-fail was found). In many cases, after a soft-fail, RGIFE is able to produce smaller models with higher predictive capacity (e.g. iteration 37 on Run 1 and iteration 19 on Run 2 for *Breast*). Figure 4 also helps in highlighting the importance of restoring blocks of attributes back after an unsuccessful trial, which is an integral and novel part of RGIFE, but not used in similar methods such as SVM-RFE. Across iterations, in both datasets, it is noticeable the presence of many blue squares indicating an increase of performance when the removed attributes were not the last ranked (lowest attribute importance). Most of the methods based on an iterative reduction paradigm only remove the bottom ranked features, however, as shown in Fig. 4, discarding higher ranked features might lead to a better predictive model (e.g. iteration 14 on Run 1 for *Breast*). The examples provided in this section emphasise the role played by two of the main novel features introduced by the RGIFE heuristic: a) the relevance of placing back features whose removal negatively affects the overall performance and b) the importance of the soft-fail and its ability to drive the reduction process towards an easier and simpler solution.

### Biological significance of the signatures

When feature selection is applied to -omics data, with the aim of discovering new biomarkers, the signature not only has to be small and highly predictive, but it also needs to contain relevant features. In our analysis, dealing with cancer transcriptomics datasets, we assessed whether the selected genes are relevant in a disease/biological context. We conducted this analysis using the *Prostate-Singh* [30] dataset as a case study. In the first part of this section, we will compare RGIFE with the other methods, while later on, we will focus on the signature generated by RGIFE-Union (the best performing policy). The signatures identified by each method are available in Section 4 of the Additional file 1.

#### Gene-disease association analysis

**Fig. 5:**
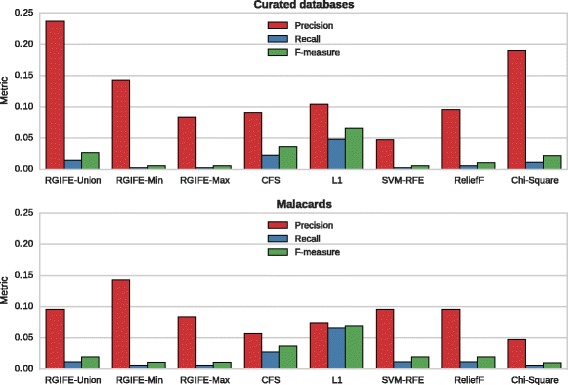
Analysis in a disease-context of the signatures selected by different methods. Two sources for the gene-disease associations were used. Each metric is referred to the number of disease-associated genes available in the signatures

In order to assess the relevance of the signatures in a disease context, we checked how many genes are already known to be important in prostate cancer. Using two different sources of gene-disease associations, we calculated: precision, recall and F-score. The higher those metrics are, the better a feature selection algorithm performs as it is able to identify the disease-associated genes from the large set of original attributes. Figure 5 shows the performances for all the signatures generated in the case study.

When using the curated sources for the associations, RGIFE-Union had the higher precision followed by Chi-Square and RGIFE-Min. The other methods performed similarly except for SVM-RFE. High precision means that several genes selected by RGIFE-Union are already known to be relevant in prostate cancer. The recall was in general low for every method and was the highest for the L1-based feature selection (which also generates the largest signatures). Likewise, L1 -selected attributes provided the highest F-measure, while similar values emerged for CFS and RGIFE-Union. The remaining approaches all scored low values. Using Malacards, compared to the curated associations, we noticed higher precision for SVM-RFE, while similar (RGIFE-Min and ReliefF) or worse performances for the other methods. An important decrease was noticed for the L1-based feature selection and Chi-Square. Recall and F-measure did not vary a lot. In general, our policies tended to have higher or similar precision than the compared methods. RGIFE-Union provided overall the best results outperforming SVM-RFE, ReliefF and Chi-Square, its signature had higher precision than CFS and L1 that, on the other hand, provided higher recall (helped by a large number of selected attributes) and F-measure.

#### Genomic alteration of the signature in independent datasets

**Fig. 6:**
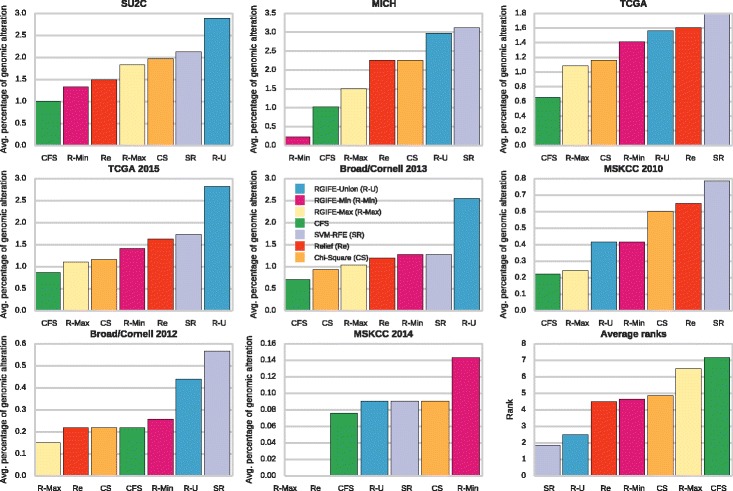
Normalised genomic alteration percentages of the signatures inferred for the case study. The alterations refer to the samples available from eight prostate cancer related datasets. The *bottom-right plot* shows the average ranks across the datasets. Higher rank indicates higher average alterations. The abbreviations and the colors for the plots are defined in the legend of the central subplot

We checked whether the genes selected by each method are relevant in prostate cancer-related data not used during the learning process. We analysed the genomic alterations of each signature in eight independent data. The average alterations are reported, dataset by dataset, in Fig. 6. The L1-based feature selection method was excluded from this analysis as it generated a signature larger than the limit of 100 genes allowed for the queries in cBioPortal. The methods are ranked by increasing percentage of alteration (the same colours are used in different datasets). The bottom-right plot shows the average rank of each method across all the datasets (higher rank means higher alteration). The two methods selecting genes that are highly altered in independent data are SVM-RFE and RGIFE-Union, with the last one clearly outperforming the others in *SUC2*, *TGCA 2015* and *Broad/Cornell 2013*. Among the other algorithms RGIFE-Max and CFS perform quite badly, overall they are the bottom ranked, while the remaining methods obtained similar performances. Those results show that RGIFE-Union selects genes that are not only highly predictive in the analysed dataset but also are highly altered in datasets, containing samples that are affected by the same disease, not used during the learning process.

In the next sections, we will focus on the analysis of the signature generated by the RGIFE-Union policy (the best performing). The signature consists of 21 genes: *ANXA2P3, TGFB3, CRYAB, NELL2, MFN2, TNN, KIAA1109, PEX3, ATP6V1E1, HPN, HSPD1, LMO3, PTGDS, SLC9A7, SERPINF1, KCNN4, EPB41L3, CELSR1, GSTM2, EPCAM, ERG*.

#### Gene-disease association from the specialised literature

We verified if the specialised literature already associates the genes in our signature with prostate cancer. Interestingly, we found that many of them are already related to prostate cancer. Just to cite few examples:

- *NELL2* is an indicator of expression changes in prostate cancer samples [41], it also contributes to alterations in epithelial-stromal homeostasis in benign prostatic hyperplasia and codes for a novel prostatic growth factor [42].
- *ANXA2P3* (annexin II) was differentially expressed in prostate carcinoma samples from USA and India [43].
- *TGFB3* is expressed in metastatic prostate cancer cell lines and induces the invasive behaviour in these cells [44].
- *CRYAB* expression values can be used to discriminate between cancerous and non-cancerous prostatic tissues [45].
- *HSPD1* was part of a four gene expression signature to detect Gleason grade 3 and grade 4 cancer cells in prostate tissue [46].
- *EPB41L3* has a potential role as a target for treatment of advanced prostate cancer [47].

In addition, we performed an enrichment analysis of the RGIFE-Union signature. The enrichment analysis is a statistical-based method to assess if a set of genes shares common biological characteristics. For this analysis we employed the PANTHER classification system [48] and its pathways knowledge base, that is a set of 176 primarily signalling pathways. Four pathways resulted statistically (confidence value of 0.05) enriched in the signature: *Heterotrimeric G-protein signalling pathway-rod outer segment phototransduction* (P00028), *B cell activation* (P00010), *T cell activation* (P00053), *Heterotrimeric G-protein signalling pathway-Gi alpha and Gs alpha mediated pathway* (P00026). Their role in prostate cancer was found to be relevant from the specialised literature. In particular, the family of heterotrimeric proteins is involved in prostate cancer invasion [49] and the (G protein)-coupled receptors (GPCRs) may contribute to tumour growth [50]. B-cells are increased in prostate cancer tissues according to a study by Woo et al. [51] and lymphotoxin derived by those cells can promote castration-resistant prostate cancer [52]. Finally, chimeric antigen receptor-engineered T cells have the potential to be used for the treatment of metastatic prostate cancer [53].

#### Signature induced network

As a further biological validation of our approach, we analysed a signature induced network (see the “Methods” section for details). The network generated using the RGIFE-Union signature resulted in 93 nodes and 190 edges (shown in Section 5 of the Additional file 1). We checked if this network is enriched for some biological knowledge using two different tools: ClueGO [54] and EnrichNet [55].

##### ClueGO

**Fig. 7:**
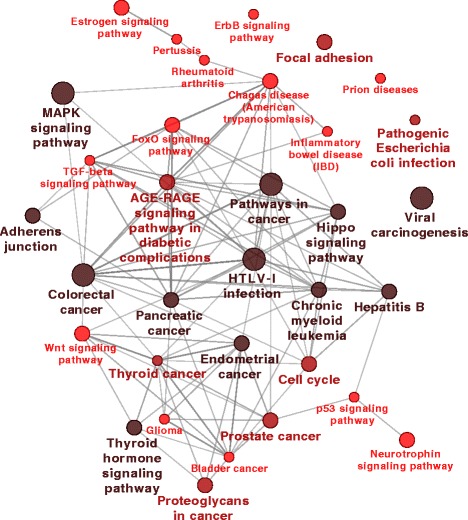
Graphical visualisation of the enriched terms (KEGG patwhays found by *ClueGO*) associated to the signature induced network nodes. Edges represent the relationship between terms based on their shared genes. The size of the node indicates the enrichment significance, the color gradient is proportional to the genes associated with the term. Only terms enriched with *p*-value < 0.05 are shown

is a Cytoscape plug-in that visualises the non-redundant biological terms for groups of genes in a functionally grouped network. KEGG pathways were used as the biological knowledge base. The results of the enrichment analysis for the nodes of the signature induced network is shown in Fig. 7; we show only pathways that are statistically enriched (*p*-value < 0.05). The edges between nodes represent the relationship between terms based on their shared genes. The size of the node reflects the enrichment significance of the node, while the colour gradient shows the gene proportion of each cluster associated with the term. One of the highest enriched terms is *pathways in cancer*, this further confirms the role of the selected genes in a cancer context. Among many cancer-related terms, we highlight the presence of the *prostate cancer* pathway as the signature was inferred from prostate cancer data. Finally, *MAPK* is a further pathway already associated, in the literature, with prostate cancer [56].

##### EnrichNet

We also validated the signature induced network with an enrichment tool that uses a different approach than ClueGO. EnrichNet is a gene set analysis tool that combines network and pathway analysis methods. It maps gene sets onto an interaction network and, using a random walk, scores distances between the genes and pathways (taken from a reference database). The XD-score is a network-based association score relative to the average distance to all pathways. The list of pathways with a statistically significant XD-score (using the STRING network as background PPI) is reported in Table 8. Several types of cancer are associated with the induced network, among them it appears *colorectal cancer* that, according to [31] Malacards, is linked with prostate cancer. Within the list of terms with a significant overlap with the pathways (not reported here) also emerges the *prostate cancer* pathway.

**Table 8:** Enriched KEGG pathways (with statistically significant XD-score) identified by *EnrichNet*

Pathway/process
hsa05210:Colorectal cancer
hsa05216:Thyroid cancer
hsa05213:Endometrial cancer
hsa05212:Pancreatic cancer
hsa05219:Bladder cancer
hsa05220:Chronic myeloid leukemia
hsa04520:Adherens junction
hsa05130:Pathogenic Escherichia coli infection
hsa05020:Prion diseases

## Discussion

In this paper, we have thoroughly studied RGIFE: a ranked guided iterative feature elimination heuristic that aims to select a small set of features that are highly predictive. Three main features differentiate RGIFE from many algorithms based on the iterative reduction paradigm: 1) the presence of a back-tracking step that allows to “place back” the features if their removal causes a decrease in the classification performance, 2) the optimal number of selected features is automatically identified rather than being specified up-front and 3) the presence of soft-fails (iterations accepted if the classification performance drop within a tolerance level). To cope with the stochastic nature of RGIFE, we defined three different policies (RGIFE-Union, RGIFE-Min and RGIFE-Max) to select the final signature.

The presented heuristic is an improvement of the method proposed in [13]. Therefore, a natural step was the comparison of RGIFE with its original version. The changes implemented in this new version include: a different base classifier, a new dynamic selection of the number of attributes to remove at each iteration, a more robust validation scheme to be used within the heuristic and the usage of optimisation strategies to reduce the likelihood to obtain local optimum solutions. The new version outperformed the original methods, using ten cancer-related datasets, in terms of: prediction accuracy, number of selected attributes and computational time. The first two improvements are likely the results of both the new relative block size approach and the extended search and usage of soft-fails. With these new strategies, the heuristic performs a less greedy search and is more prone to deal and analyse smaller sets of data; all together these approaches led to better and simpler solutions. The decrease of the computational time is due to the adoption of a faster base classifier, from BioHEL, an evolutionary learning classifier, to a random forest.

Afterwards, the ability of RGIFE to identify relevant attributes was tested using many different synthetic datasets. RGIFE-Union clearly outperformed the other five feature selection methods in the analysis of synthetic datasets with different characteristics: noise, nonlinearity, correlation, etc. The heuristic was proven good in selecting relevant features while discarding irrelevant information. The other two policies performed slightly worse but in line with the other methods. When analysing datasets that aim to reflect the problematic of microarray data, opposite results were obtained. Compared with CFS and L1-based feature selection, the RGIFE policies constantly selected fewer relevant attributes (up/down-regulated genes), while producing a perfect classification, when applied to the *madsim* data [28]. On the other hand, poorer performance were obtained by RGIFE on the SD datasets [26]. The bad results of RGIFE are likely to be associated with the low number of samples (25 for each of the three classes) available in the SDs data. When dealing with only a few samples, both the predictive performance and the attribute importance, used by RGIFE to evaluate feature subsets, become unstable. Noisy attributes are misinterpreted as relevant and eventually are chosen in the final solution. In addition, the problem of the small number of instances was also amplified by the double cross-fold validation: external to assess the performance of each method and internal within RGIFE to estimate the goodness of the feature sets. However, quite interesting, the accuracy provided by the RGIFE selected attributes, when determined with a random forest and a Gaussian Naive Bayes classifier, was the best for SD2 and SD3.

Encouraged by the good results obtained with synthetic data, we evaluated and compared our heuristic using ten cancer-related transcriptomics datasets. With the Friedman test, we assessed that there is no statistical difference between the predictive performance of the methods (except for RGIFE-Min that was statistically worse than ReliefF when evaluated with the random forest). While RGIFE-Union and RGIFE-Max had similar results, RGIFE-Min clearly performed quite badly. The reason behind this poor behaviour probably relies on the extremely small number of selected attributes: up to 15 and 18 times lower if compared with RGIFE-Max and RGIFE-Union respectively. When we contrasted the number of attributes chosen by the RGIFE policies, CFS and the L1-based feature selection, a clear difference emerged. All our methods always selected fewer features than the other two approaches. For RGIFE-Max and RGIFE-Min this difference was statistically significant. Overall, from a computational point of view, we propose a heuristic for the identification of biomarkers that: 1) performs as good as the compared feature selection methods, but 2) consistently selects fewer attributes.

The second part of the validation was focused on the analysis of the selected genes in a biological context. To perform a detailed validation, we used a prostate cancer dataset as a case study. The specialised literature supports the evidence that the genes chosen by RGIFE-Union (best performing policy) are relevant in prostate cancer. In addition, when tested with PANTHER, the signature was shown to be enriched in four biological pathways, all of them associated, from specialised publications, to prostate cancer. The relevance in a disease context was further confirmed when we used the gene-disease association knowledge retrieved from different sources. The signatures identified by RGIFE contain several genes already known to be associated with prostate cancer, confirming the ability of the proposed approach to choose relevant features. It is well known that multiple optimal solutions can be identified when generating predictive models from high dimensional -omics data [18], however among them, RGIFE tends to prefer solutions having more relevant genes in a disease context (when compared with the tested methods). The analysis of the genomic alterations in independent tumour samples showed that RGIFE-Union and SVM-RFE select genes that are highly altered in datasets where the samples are affected by the same disease. A high average alteration in samples not used for the learning process demonstrates that RGIFE selects genes that are not exclusively tight to the dataset analysed during the reduction phase, but that are potentially informative for the disease. Finally, the signature induced network (from a PPI network) was shown to be compact (small diameter) and enriched for many biological pathways related to cancer and in particular to prostate cancer. This type of network-based analysis suggests that genes involved in a specific disease tend to be highly connected and separated, between each other, by a low degree (the network was induced calculating all the shortest paths between the signature genes). Overall, the case study assessed that RGIFE extracts genes that: 1) share common biological characteristics and are enriched for pathways important from a clinical (prostate cancer) point of view, 2) are relevant in a disease context according to both the specialised literature and two different databases of gene-disease associations and 3) are highly altered in related independent datasets not used for the signature identification process.

All in all, the presented heuristic is a useful and powerful tool for the identification of small predictive sets of biomarkers. According to the analysis performed, using multiple criteria, RGIFE-Union seems to be the best RGIFE policy leading to important predictive performance and relevant biomarkers. However, RGIFE-Min could be applied when willing to trade a small drop of performances in favour to a significant smaller set of biomarkers that could be more easily verified with in vitro/in vivo experiments. RGIFE-Min predictive performance were found statistically worse only when using the random forest. In addition, its Success Index was in some instances higher than the compared methods (fifth on average, in line with the performance of the L1-based feature selection method). Finally, the RGIFE-Min ability of identifying few but relevant attributes was confirmed by the highest precision obtained when using Malacards gene-disease associations, and by the forth average rank in the independent data analysis (in line with ReliefF performance).

One of the main advantages of RGIFE is its extreme flexibility in terms of attributes ranking and estimation of the predictive power of the biomarker signatures. In this paper, we used a random forest classifier coupled with the gini impurity as metric for the feature importance, however, many other classification algorithms can be employed, if able to provide a variable ranking based on the generated models. In fact, it is easy to switch from a random forest to a single decision tree or to a gradient boosting classifier (as all of these are implemented within *scikit-learn* and provide an attribute importance score). According to this observation, in future, we would like to test the performances of different classifiers and feature rankings approaches. In addition, trying to tackle the problematic encountered when analysing the SD datasets, we would like to test other internal strategies to evaluate the goodness of the attribute tests (e.g. different *k* values for the *k*-fold cross-validation), so that RGIFE can well perform even with datasets containing only a few samples per class. It would also be interesting to apply RGIFE to regression problems (e.g. time-series data). Furthermore, we would like to assess if RGIFE can benefit from the usage of a fixed attribute ranking as suggested by [57].

## Conclusions

In this paper we present a heuristic for the identification of small sets of highly predictive biomarkers called RGIFE. RGIFE is guided, in its search for an optimal solution, by the information extracted from machine learning models when solving a classification task. The presented work is a substantial extension of the original method proposed by Swan et. al [13]. We assessed that the features introduced in the new version of RGIFE provide higher performance in terms of: a) prediction accuracy, b) size of the selected signatures and c) computational time. When RGIFE was compared with methods commonly employed to solve the task of biomarker identification, using synthetic datasets, it showed better ability in identifying relevant attributes and discard irrelevant information for most of the tested datasets. The comparison performed by employing ten cancer-related transcriptomics datasets revealed that RGIFE provided (statistically) similar prediction performance while consistently using fewer attributes. More important, from a biological and clinical point of view, the biomarkers selected by RGIFE were mostly already known to be relevant in a disease context (using prostate cancer as a case study) and showed a consistent pertinence on eight different independent datasets. Overall, we propose a robust and flexible heuristic that performs well if coupled with different classifiers and is capable of extracting, among a large set of attributes, highly predictive and disease relevant biomarkers.

## Additional file

Additional file 1 Supplementary material. Supporting information referenced in the main text. (PDF 464 kb)

## Abbreviations

DB-SCV: Distributed-balanced stratified cross validation
FCR: Full class relevant
GNB: Gaussian naive bayes
GPCRs: G-protein coupled receptors
KNN: K-nearest neighbours
PCR: Partial class relevant
PPI: Protein-protein interaction network
RF: Random forest
RGIFE: Ranked guided iterative feature elimination
SVC: Support vector classifier
