# Supplementary material for: RGIFE: a ranked guided iterative feature elimination heuristic for the identification of biomarkers
Source: BMC Bioinformatics. 2017 Jun 30;18:322. doi: 10.1186/s12859-017-1729-2 (PMC5493069; doi:10.1186/s12859-017-1729-2)
Supplement: Supplementary file 1 — Supplementary material. Supporting information referenced in the main text. (PDF 464 kb) [file 12859_2017_1729_MOESM1_ESM.pdf]

Supplementary Material

RGIFE: a ranked guided iterative feature elimination heuristic for the identification of biomarkers

Nicola Lazzarini and Jaume Bacardit

1 Predictive performance of Random Forest and BioHEL

The new presented version of RGIFE replaces BioHEL Bacardit *et al.* (2009) with a random forest Breiman (2001) as base classifier. This choice is mainly due to reduce the overall computational cost required by the heuristic. In order to check whether the usage of a different base classifier can drastically affect the overall performance of the heuristic, we tested the predictive performance of BioHEL and random forest using the 10 transcriptomics datasets (considering the whole set of attributes) presented in the main manuscript. We calculated the accuracy obtained by each classifier using a 10-fold cross-validation. The accuracies, dataset by dataset are reported in Table S1.

**Dataset**	**Random Forest**	**BioHEL**
CNS	0.637	0.645
Leukemia	0.986	0.946
Breast	0.860	0.877
Dlbcl	0.597	0.553
Prostate-Singh	0.913	0.914
Prostate-Sbo.	0.740	0.749
Pancreas	0.898	0.873
AML	0.687	0.663
Colon-Breast	0.947	0.927
Bladder	0.806	0.800

Table S1: BioHEL and Random Forest classification accuracy for each dataset, in 10-fold cross-validation experiments on the original set of attributes.

Using the Wilcoxon rank-sum statistic test we established that the performances of the two classifier are statistically equal. In fact, on average the difference in accuracies is only 0.016 in favor of the random forest.

2 Time complexity

We tested the time complexity of each feature extraction method across 10 different datasets. We calculated the time, measured in second, required to identify the optimal subset of features by each method presented in the main manuscript. Figure S1 shows the running times averaged across the experiments performed for the 10-fold cross-validation. When plotting the times required by RGIFE, we considered, for each fold, the average time obtained by three executions of the heuristic. Overall, the methods more time consuming are CFS and RGIFE, they performed similarly with large datasets (in Figure S1 the datasets are ranked by increasing number of total attributes), while RGIFE was clearly slower for smaller dataset. The other four methods in general required less computational time with the L1-based approach that appeared to be the fastest one.

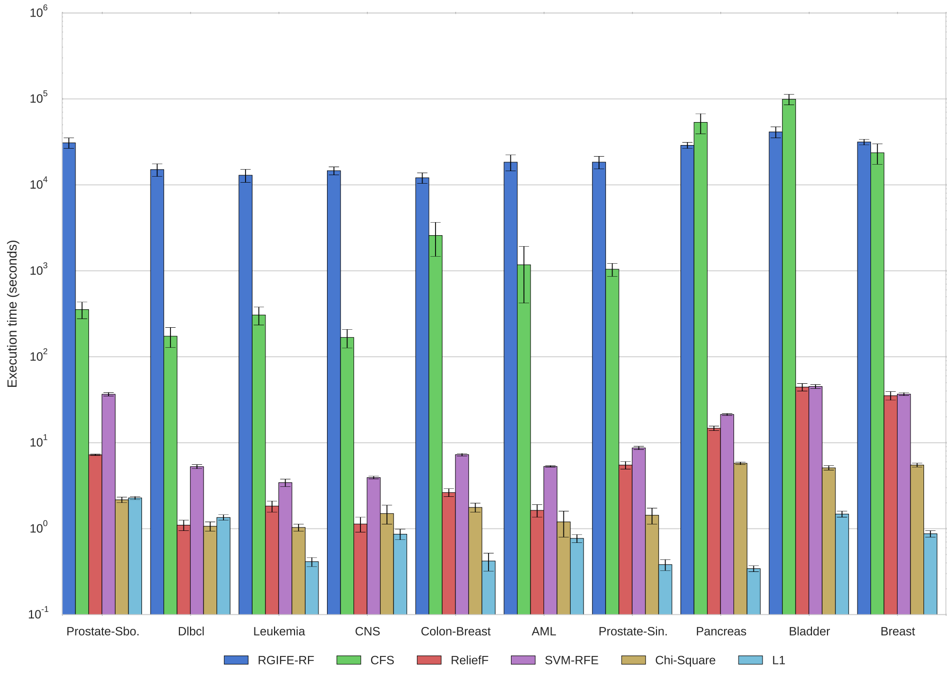

Figure S1: Average execution times (calculated using a 10-fold cross-validation) of each methods across different datasets. The datasets are sorted by increasing number of attributes.

3 Default parameter values of the methods used for the analysis

This section includes the default parameters used for the methods employed in the analysis. The WEKA software (Hall *et al.*, 2009) (version 3.6.10) was used for the implementation of CFS, SVM-RFE, ReliefF and Chi-Square, while the *sci-kit learn* python library (Pedregosa *et al.*, 2011) (version 0.17.1) was used for the L1-based feature selection.

- **RGIFE**:
  - Random Forest depth: unlimited
  - Random forest trees: 3000
  - CV scheme: DB-SCV
  - CV repetitions: 10
- **CFS**:
  - Search method: Best First
  - Search direction: forward
  - Search termination: 5
- **SVM-RFE**:
  - SVM kernel: linear
  - Complexity: 0
  - Epsilon: 1.0E-25
  - Percent threshold: 0
  - Percent to eliminate per iteration: 5
  - Tolerance: 1.0E-10
- **ReliefF**:
  - Number of neightbours: 10
  - Sigma: 2
  - Sample size: all
- **Chi-Square**:
  - Missing merge: True
- **L1-based feature selection**:
  - SVC kernel: linear
  - Cost: 1
  - Penalty: L1
  - Loss: Squared hinge
  - Tolerance: 1.0E-4
  - Dual opt. problem: True

4 Predictive performance with synthetic datasets

We tested the predictive performance of the attributes selected by each method using different synthetic datasets. Table S2 shows the accuracies, obtained from a 10-fold cross-validation, using the datasets described in Bolón-Canedo *et al.* (2013). In Table S3 are reported the accuracies calculated from the analysis of the *madsim* data Dembélé (2013). Each row includes the average values associated to the analysis of datasets having 1%, 2% and 5% of up/down regulated attributes (genes).

Class.	Dataset	RGIFE-Min	RGIFE-Max	RGIFE-Union	CFS	Relief	SVM-RFE	Chi-Square	L1
RF	**CorrAL**	0.675	0.725	0.758	0.675	0.758	**0.783**	0.658	0.733
	**XOR-100**	**1.000**	**1.000**	**1.000**	0.500	0.480	0.500	0.420	0.580
	**Parity3+3**	**1.000**	**1.000**	**1.000**	0.521	0.933	0.429	0.474	0.502
	**Monk3**	**0.935**	**0.935**	**0.935**	**0.935**	0.910	N/A	**0.935**	**0.935**
	**Madelon**	0.869	0.868	**0.874**	0.805	0.866	0.787	0.835	0.744
	**SD1**	0.240	0.319	0.333	0.414	0.452	**0.478**	0.437	0.421
	**SD2**	0.389	**0.639**	0.635	0.521	0.456	0.466	0.477	0.458
	**SD3**	0.317	**0.626**	**0.626**	0.428	0.476	0.473	0.487	0.526
SVM	**CorrAL**	0.633	0.625	0.658	0.608	0.642	**0.725**	0.600	0.658
	**XOR-100**	0.598	0.700	**0.707**	0.500	0.400	0.480	0.500	0.360
	**Parity3+3**	0.348	0.348	0.348	**0.550**	0.319	0.502	0.500	0.505
	**Monk3**	0.828	0.828	0.828	0.813	0.820	N/A	**0.837**	0.789
	**Madelon**	0.598	**0.600**	**0.600**	0.557	**0.600**	0.593	0.595	0.562
	**SD1**	0.238	0.293	0.281	**0.437**	0.386	0.376	0.369	0.398
	**SD2**	0.371	0.349	0.351	0.395	0.626	0.459	0.473	0.473
	**SD3**	0.306	0.358	0.393	0.353	0.469	0.461	0.492	**0.515**
KNN	**CorrAL**	0.575	0.600	0.625	**0.758**	0.733	**0.758**	0.625	0.608
	**XOR-100**	**0.987**	0.962	0.973	0.560	0.460	0.460	0.500	0.520
	**Parity3+3**	0.219	0.219	0.219	0.550	**0.936**	0.486	0.560	0.543
	**Monk3**	0.887	0.887	0.887	**0.902**	0.894	N/A	0.877	0.878
	**Madelon**	0.698	0.694	0.699	0.868	**0.913**	0.828	0.894	0.805
	**SD1**	0.292	0.350	0.352	0.423	**0.453**	0.442	0.414	0.374
	**SD2**	0.436	0.393	0.419	0.421	**0.487**	0.470	0.476	0.446
	**SD3**	0.352	0.375	0.441	0.462	0.510	0.545	0.520	**0.546**
GNB	**CorrAL**	0.608	0.600	0.633	0.650	0.708	**0.717**	0.600	0.683
	**XOR-100**	0.602	0.689	**0.691**	0.480	0.420	0.480	0.480	0.420
	**Parity3+3**	**1.000**	**1.000**	**1.000**	0.567	0.233	0.486	0.500	0.488
	**Monk3**	**0.894**	**0.894**	**0.894**	0.887	0.887	N/A	**0.894**	0.887
	**Madelon**	0.698	0.694	0.699	0.699	**0.703**	0.688	0.699	0.675
	**SD1**	0.21	0.278	0.249	0.437	0.463	**0.477**	0.411	0.382
	**SD2**	0.283	**0.666**	**0.666**	0.451	0.533	0.458	0.443	0.474
	**SD3**	0.293	**0.667**	**0.667**	0.346	0.494	0.473	0.499	0.498

Table S2: Accuracies obtained by each method across the synthetic datasets using four classifiers. The highest accuracies are shown in bold. N/A is used for SVM-RFE when tested with the *Monk3* dataset because the method can not deal with categorical attributes. RF: Random Forest, KNN: K-nearest neighbour, GNB: Gaussian Naive Bayes.

Class.	Attributes	RGIFE-Min	RGIFE-Max	RGIFE-Union	CFS	Relief	SVM-RFE	Chi-Square	L1
RF	**5 000**	0.997	0.997	**1.000**	**1.000**	**1.000**	**1.000**	**1.000**	**1.000**
	**10 000**	0.977	0.980	**1.000**	**1.000**	**1.000**	**1.000**	**1.000**	**1.000**
	**20 000**	0.993	0.993	**1.000**	**1.000**	**1.000**	**1.000**	**1.000**	**1.000**
	**40 000**	0.983	0.993	**1.000**	**1.000**	**1.000**	**1.000**	0.997	**1.000**
SVM	**5 000**	0.990	0.987	**1.000**	**1.000**	**1.000**	**1.000**	**1.000**	**1.000**
	**10 000**	0.983	0.987	**1.000**	**1.000**	**1.000**	**1.000**	**1.000**	**1.000**
	**20 000**	0.990	0.990	**1.000**	**1.000**	**1.000**	**1.000**	**1.000**	**1.000**
	**40 000**	0.987	0.993	**1.000**	**1.000**	**1.000**	**1.000**	0.997	**1.000**
KNN	**5 000**	0.997	0.997	**1.000**	**1.000**	**1.000**	**1.000**	**1.000**	**1.000**
	**10 000**	0.977	0.987	**1.000**	**1.000**	**1.000**	**1.000**	**1.000**	**1.000**
	**20 000**	0.987	0.990	**1.000**	**1.000**	**1.000**	**1.000**	0.997	**1.000**
	**40 000**	0.997	0.987	**1.000**	**1.000**	**1.000**	**1.000**	**1.000**	**1.000**
GNB	**5 000**	0.997	**1.000**	**1.000**	**1.000**	**1.000**	**1.000**	**1.000**	**1.000**
	**10 000**	0.983	0.993	**1.000**	**1.000**	**1.000**	**1.000**	**1.000**	**1.000**
	**20 000**	0.990	0.993	**1.000**	**1.000**	**1.000**	**1.000**	**1.000**	**1.000**
	**40 000**	0.987	0.997	**1.000**	**1.000**	**1.000**	**1.000**	0.997	**1.000**

Table S3: Accuracies obtained by each method across the synthetic datasets using four classifiers. The highest accuracies are shown in bold. RF: Random Forest, KNN: K-nearest neighbour, GNB: Gaussian Naive Bayes.

5 Signatures analysed in the case study

In here we report the signatures (list of genes) extracted by each method when analysing the Prostate-Singh (Singh *et al.*, 2002) dataset within the case study.

RGIFE-Min: EPB41L3, HPN, HSPD1, PTGDS, NELL2, TGFB3, GSTM2

RGIFE-Max: TNN, KCNN4, CELSR1, KIAA1109, PEX3, HPN, MFN2, ATP6V1E1, HSPD1, PTGDS, SLC9A7, NELL2

RGIFE-Union: ANXA2P3, TGFB3, CRYAB, NELL2, MFN2, TNN, KIAA1109, PEX3, ATP6V1E1, HPN, HSPD1, LMO3, PTGDS, SLC9A7, SERPINF1, KCNN4, EPB41L3, CELSR1, GSTM2, EPCAM ERG

SVM-RFE: HPN, HSPD1, MAF, S100A4, JUNB, SERPINB5, C7, TBC1D2B, SDC1, IPO5, SFRP1, PGCP, PEX3, SPTB, FOXO1, GSTA4, CD38, RBBP6, SERINC5, VCAN, C5orf13

Relief: HPN, HSPD1, NBL1, MAF, DPYSL2, C7, PEX3, TGFB3, CFD, TARP, PAGE4, XBP1, PTGDS, PDLIM5, RBP1, LMO3, SERPINF1, DPT, FAM107A, SERINC5, TACSTD2

Chi-Square: HPN, NELL2, HSPD1, RBP1, PTGDS, CALM1, CDKN1C, PDLIM5, CFD, SERPINF1, TARP, COX7A1, GSTM2, CRYAB, RPLP0, TGFB3, ANGPT1, EPCAM, VCL, TMSB15A, LMO3

CFS: RPL13, RPLP0, HBB, RPL6, HOXB3, TSPAN2, MCF2L2, PHEX, CNKSR2, CPA3, PLA2G7, SCGN, COL13A1, CHD9, EPB41L3, MEIS2, CREB3L1, ZFP161, ADORA2A, GLCE, SLC35A2, DDHD2, WIF1, HEPH, TMSB15A, DIXDC1, KIAA0427, PEX3, ZNF146, TRIM23, HPN, PITX1, SLC1A1, PENK, RBP1, C14orf2, TUBB2A, MAP1LC3B, CALCOCO2, CYP1B1, SLC25A6, ORAI2, GSTA4, AHR, SERPINF1, COBLL1, STK38L, SLC7A5, MRPL40, DST, JUNB, GSTP1, LGALS1, SPTAN1, ABI1, SPON1, ROCK2, AKR1B1, TSC22D3, GPM6A, PLAGL1, PLA2G2A, CKS1B, PDLIM5, HSPD1, LMO3, S100A4, PKD2, PTGDS, CDKN1C, CRMP1, CFD, CALR, NELL2, RGS10, ABL1, SERINC5, PMS2L5, MAPK10, GTF2B, RGN, ERG, SERPINB5, NAP1L3, LAMB1, GSTM2, IL11RA, CYP21A2

L1-based: AVPR1B, TGM2, TSC22D3, ACTG1, ACTG2, MYH11, LYPLA2, BGN, HBB, SBF1, B2M, PRB1, MROH5, IGKC, CLSTN1, MYL9, ST5, GRK6, GADD45B, LYZ, PTGER3, ANXA2P3, PTP4A3, EDN2, ZNF337, MSMB, IFITM3, P4HB, SLC25A6, IFI30, ATP1B1, KLK2, KLK3, RPL10, RPL13, CYP3A5, COX6A1, RPL19, LOC91316, ORM1, NME2, CCND1, SFI1, SFN, NPY, UBB, MAF, ACTB, ACTA2, GRIN2C, RPL8, RPL9, HLA-C, PABPC1, RPL5, GAPDH, SEPT9, TUBB4B, NDRG1, PAGE4, RPS2P5, C21orf2, UBE3B, NBL1, ZFP36, MT1H, C4A, TACSTD2, MT1G, C1QL1, NACA, TPT1, FOS, VCL, UBC, IGL@, IGFBP5, COX7A1, FTO, LGALS3BP, PMP22, ALDH4A1, SDC1, KRT17, KRT15, KRT13, FLNA, LUZP1, CCL2, RPLP1, RPLP0, RPL18A, RPS6, RPS3, TXNIP, RPS17, LUM, TMED2, RPL6, TPM1, RPL13A, FASN, RPL7, CST3, DUSP1, TNFRSF6B, MARCKSL1, RPS24, ZFP36L1, ZFP36L2, TOP3B, PLA2G2A, LTF, S100A4, RPS4X, CLU, LRP3, HDGF, ACPP, RPSA, C7, GSTM2, ID1, CTGF, HSP90AA1, PSCA, COX7C, RPL36A, RBM3, RPS14, TMSB4X, EEF1A1, JUNB, JUND, TARP, ATP11A, PTGDS, XBP1, HLA-DRA, SERPINA3, RPL29, CEBPD, HSPD1, LDHA, AMD1, GALNS, PDIA2, IGH@, AAK1, ARR3, HPN, AP2A2, IGHM, VAMP1, SORD, E2F4, HAP1, C1QTNF3, CFD, RPL32, MAP3K11, GSTP1, TSPAN1, PTRF, SYN1, EEF2

6 Signature induced network

We generated a signature induced network from a PPI network by aggregating all the shortest paths between all the genes extracted by RGIFE-Union when applied to case study dataset (Singh *et al.*, 2002). If multiple paths existed between two genes, the path that overall (across all the pairs of genes) was the most used was included. The paths were taken from the PPI network employed in (Vlassis and Glaab, 2015) that was assembled from 20 public protein interaction repositories (BioGrid, IntAct, I2D, TopFind, MolCon, Reactome-FIs, UniProt, Reactome, MINT, InnateDB, iRefIndex, MatrixDB, DIP , APID, HPRD, SPIKE, I2D-IMEx, BIND, HIPPIE, CCSB), removing non-human interactions, self-interactions and interactions without direct experimental evidence for a physical association. The network resulted in 93 nodes and 190 edges is illustrated in Figure S2.

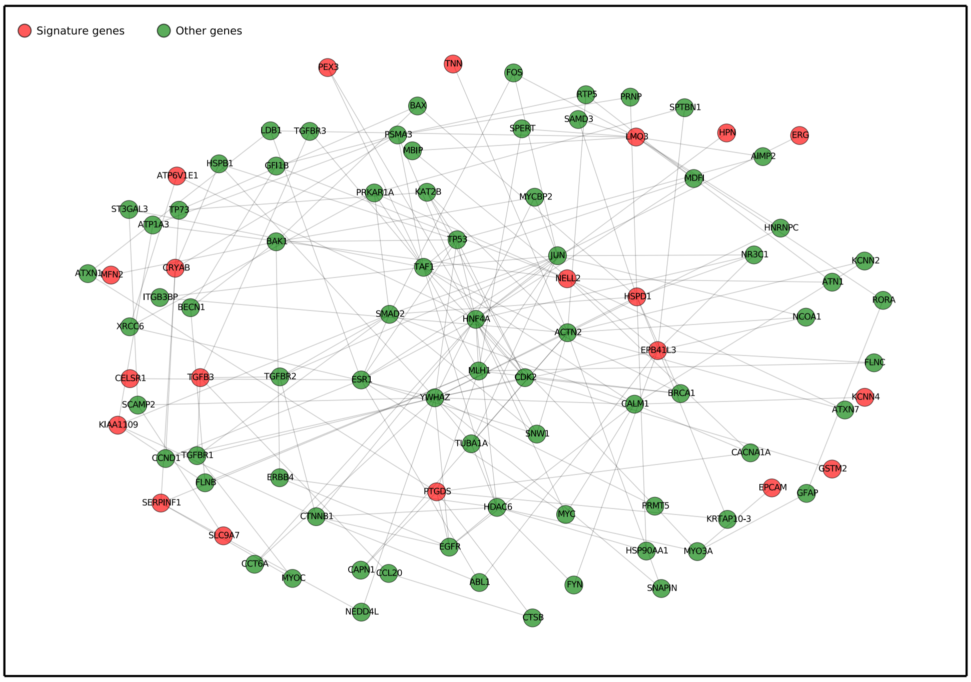

Figure S2: Signature induced network generated from the genes extracted using the RGIFE-Union policy.

References

Bacardit, J. et al (2009). Improving the scalability of rule-based evolutionary learning. *Memetic Computing*, **1**(1), 55–67.

Bolón-Canedo, V. et al (2013). A review of feature selection methods on synthetic data. *Knowledge and Information Systems*, **34**(3), 483–519.

Breiman, L. (2001). Random forests. *Machine Learning*, **45**(1), 5–32.

Dembélé, D. (2013). A flexible microarray data simulation model. *Microarrays*, **2**(2), 115–130.

Hall, M. et al (2009). The weka data mining software: An update. *SIGKDD Explor. Newsl.*, **11**(1), 10–18.

Pedregosa, F. et al (2011). Scikit-learn: Machine learning in Python. *Journal of Machine Learning Research*, **12**, 2825–2830.

Singh, D. et al (2002). Gene expression correlates of clinical prostate cancer behavior. *Cancer Cell*, **1**(2), 203 – 209.

Vlassis, N. et al (2015). Genepen: analysis of network activity alterations in complex diseases via the pairwise elastic net. *Statistical applications in genetics and molecular biology*, **14**(2), 221–224.
